# A Hybrid Rule-Based and Data-Driven Approach to Illegal Transshipment Identification with Interpretable Behavior Features

**DOI:** 10.3390/s22249581

**Published:** 2022-12-07

**Authors:** Lei Deng, Yuchen Niu, Limin Jia, Wen Liu, Yu Zang

**Affiliations:** 1 State Key Laboratory of Rail Traffic Control and Safety, Beijing Jiaotong University, Beijing 100044, China; 2 School of Traffic and Transportation, Beijing Jiaotong University, Beijing 100044, China; 3 School of Information and Communication Engineering, Communication University of China, Beijing 100024, China; 4 Beijing Research Center of Urban Traffic Information Sensing and Service Technologies, Beijing Jiaotong University, Beijing 100044, China; 5 National Engineering Research Center of Transportation Safety and Emergency Informatics, China Transport Telecommunications and Information Center, Beijing 100011, China; 6 China Transport Informatics National Engineering Laboratory Co., Ltd., Beijing 100094, China

**Keywords:** transshipment, marine monitoring, unsupervised learning, knowledge-based system, marine crime, intelligent monitoring

## Abstract

Illegal transshipment of maritime ships is usually closely related to illegal activities such as smuggling, human trafficking, piracy plunder, and illegal fishing. Intelligent identification of illegal transshipment has become an important technical means to ensure the safety of maritime transport. However, due to different geographical environments, legal policies and regulatory requirements in each sea area, there are differences in the movement characteristics and geographical distribution of illegal transshipment behavior in different time and space. Moreover, in areas with dense traffic flow, normal navigation behavior can easily be identified as illegal transshipment, resulting in a high rate of misidentification. This paper proposes a hybrid rule-based and data-driven approach to solve the problem of missing identification in fixed threshold methods and introduces a traffic density feature to reduce the misidentification rate in dense traffic areas. The method is both interpretable and adaptable through unsupervised clustering to get suitable threshold distribution combination for regulatory sea areas. The evaluation results in two different sea areas show that the proposed method is applicable. Compared with other widely used identification methods, this method identifies more illegal transshipment events, which are highly suspicious, and gives warning much earlier. The proposed method can even filter out misidentification events from compared methods’ results, which account for more than half of the total number.

## 1. Introduction

With economic globalization, international trade has become an important part of the world economy, and maritime transport bears more than 80% of global trade freight [1]. Security and transparency are vital to maintaining this mode of transport, but they are threatened by illegal activities at sea such as illegal unregulated and unreported (IUU) fishing, human trafficking, smuggling, and piracy plunder [2]. A common feature of these crimes is that goods, supplies, or personnel are transferred between vessels during the voyage, which is broadly defined as transshipment behavior [3], also known as ship-to-ship transfer.

In the case of IUU fishing, fishing vessels remain at sea for long periods, fishing and waiting for transshipment vessels to land caught seafood. This leads to overfishing and allows illegally caught fish mixed with legal products together to enter the market, making it difficult to estimate the true number of fish caught in the region [4]. The Food and Agriculture Organization (FAO) of the United Nations has identified transshipment as a key indicator of international deterrents to IUU fishing [5]. For human trafficking, by receiving supplies and fuel from other ships, crew members can be kept at sea indefinitely, raising issues such as slavery and bonded labor [6]. Concerning smuggling activities, illegal goods are transshipped at sea by organized criminal networks to falsify the origin of goods and transport them unregulated across the world’s oceans. To monitor and combat these maritime illegal activities, it is crucial to identify suspected illegal transshipment behavior at sea [7].

These infringements are often committed in areas where supervision is weak, such as the high seas, and the sheer size of the world’s oceans presents a major obstacle to the implementation of maritime governance [8]. However, the trajectory of the vessels involved in transshipment has distinct characteristics that differ from most of the other normal ship movements, namely rendezvous, as shown by the ships being close to each other at low speed for a period far from strictly regulated sea areas. In contrast, other ships normally navigate along the main traffic corridors, sailing directly to their destinations and maintaining a safe distance from other vessels. To monitor the trajectory of vessels in vast ocean space, technology such as drones, imaging satellites, combined satellite-terrestrial AIS (automatic identification system) [9], and other remote sensors can be used. By comparision to traditional maritime monitoring through manned ships, these methods are cheaper and more accessible for states [10,11,12].

Even though illegal transshipment behavior has some common characteristics, it remains difficult to develop an ideal model that can automatically and accurately identify them for the following reasons. Firstly, it is difficult to determine the extent of behavior characteristics, such as speed and proximity of the vessels. Different locations, environmental conditions, and regulatory constitutions influence the characteristics of the behavior in different contexts [13] and if the ships are considered as mass points when calculating the distance between them, the actual distance will differ from the calculated one, particularly when the vessels are large. Secondly, the availability of real-world examples for the study is limited. Even in regions that have adopted transshipment regulations [14,15,16], data on transshipments is closely guarded and often considered sensitive business information. While in other regions, transshipments activites are without any independent observations, transshipped catch verification, or suspected transnational criminal activities monitoring [3]. Thirdly, the basic features of illegal transshipment are similar to certain normal navigation behaviors. In the following scenario, when a ship is traveling in a channel crowded with other ships, it chooses to travel at a slower speed for navigation safety and maintain this state while in the channel. According to its basic characteristics, the ship is likely to be misidentified as engaged in illegal transshipment.

Considering the common feature of illegal transport and meeting actual regulatory requirements for interpretability, much of the current literature uses rule-based methods, by determining whether the characteristics of two ships’ trajectories meet the predetermined patterns [3,17,18,19,20,21]. Additionally, a relatively small body of literature uses data-based methods such as machine learning techniques which learn the behavior pattern from labeled samples [22,23]. The main advantage of rule-based methods is their interpretability, which is essential for regulators to make decisions regarding suspicious or dangerous vessel activities. However, such patterns do not learn from data, and can barely identify ship behavior that fits with some priori definitions that are highly influenced by human subjectivity [24]. Although the data-based methods can straightly learn the pattern from real-world examples, they highly depend on the quality of datasets, abandon the use of expert experience, and the identification results are non-interpretable.

Defining the optimal threshold is a challenge that the rule-based approach faces. For transshipment behavior, it includes setting properly minimum encounter duration, maximum encounter speed, and maximum distance between ships. For this problem, the literature [17,21] directly uses fixed thresholds determined by experts. In [18], an interface is provided for the expert to adjust the threshold based on the recognition performance in the application. The sensitivity of each threshold is analyzed in [3], and a comparison is made between the percent change in total duration for different threshold values, and the optimal threshold is determined by finding the more tolerant boundary in the interval that triggers the biggest variation; some use the fuzzy set theory to blur pre-determined threshold boundaries [20]. While these methods can select relatively reasonable thresholds, the values of each threshold are chosen independently rather than in combination and are not fully determined by the behavior characteristics of the sea area.

Inference results being either true or false is another shortcoming of most identification methods. In this way, even a minor perturbation could result in a completely different outcome. Markov Logic Networks are used in the literature [19,20] to introduce probabilistic uncertainty into the rule base by learning weights to each formula. Thus, it does not exclude events that do not meet all the conditions in the rule, but using this probability directly as a measure of suspicion is less interpretable. In the literature [21], fuzzy logic systems have been used to first identify the transit behavior using fewer rules and then to classify the behavior suspiciously based on other features, in order to reduce missed identifications while making the level of suspicion more interpretable. However, the classification of the suspicion level only takes into account the movement features of the ship and not considering the situation of the surrounding sea.

Another problem is that illegal transshipment behavior exhibit similar characteristics to vessels that navigate in densely trafficked areas of the sea, where maritime surveillance is relatively strict. To solve this misidentification problem, various studies have attempted to allocate special areas [3,13,17], such as areas near ports or coastlines, anchorages, and major traffic corridors, where rendezvous behaviors are not considered illegal transshipment. In this way, misidentified events can be effectively removed in designated areas. However, if these areas are not accurate and comprehensive enough to include all traffic main routes, the capacity of identification ability will be greatly reduced, and the ideal shape is hard to slice out in the real world. When a large number of misidentification results are generated, genuine transshipment events will be swamped, making it difficult to apply to actual maritime surveillance.

To solve these problems, we proposed a method to identify suspected illegal transshipment by incorporating knowledge-based and data-driven methods, allowing the model to learn suitable threshold combinations of predefined rules by unsupervised learning from the vessel’s dynamic data in a specific sea area. Relatively loose rules are used for preliminary screening for suspected illegal transshipment, and the suspicion level is obtained based on clustering results of vessel motion features and a traffic density feature, which was introduced to reduce suspicion of misidentification in dense traffic areas without precisely delineating special sea areas.

The main contribution of this study are summarized as follows:

- An illegal transshipment identification scheme that can benefit from the interpretability advantages of the rule-based system while fully utilizing ship navigation data.
- A methodology for unsupervised learning appropriate threshold distributions of transshipment behavior in the monitored sea area.
- A traffic density feature was introduced to reduce the suspicion level of misidentification in dense traffic areas.
- A classification of suspicion is designed based on the actual regulatory needs and is interpretable.

The proposed method is evaluated on two navigation datasets within different sea areas: a public dataset around Brest ports in France and a dataset containing some areas with high traffic volume around the Rizhao port in China. The experiment results show our methods can achieve more accurate and comprehensive identification compares to the rule-based methods proposed in the literature [17], especially in dense traffic areas. A similar method is also used in [3] to estimate the number of transshipment events involved in IUU fishing globally.

The rest of the paper is organized as follows. Section 2 introduces the methods in this paper. The details of the experiments and results are presented in Section 3, and the results are discussed in Section 4. Section 5 concludes the paper and gives future work recommendations.

## 2. Methodology

### 2.1. Overall Methodology

**Figure 1:**
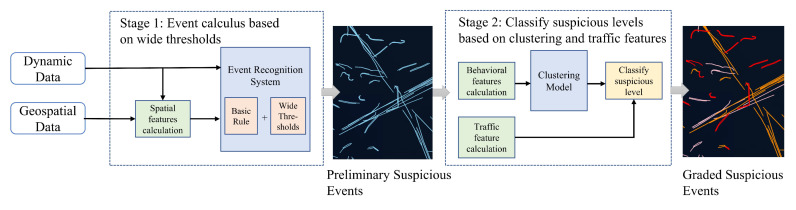
Overall methodology framework of the proposed transshipment identification framework.

The main goal of this method is to identify suspected illegal transshipments comprehensively and accurately by taking into account the behavior characteristics difference between sea areas and similarity with ships sailing in highly trafficked waters. To achieve this goal, a hierarchical identification framework is proposed. As shown in Figure 1, there are two main stages in this method. In the first stage, the system performs event inference based on basic rules and wide thresholds. In the second stage, the system classifies suspicion levels based on clustering results and the traffic density feature.

In the first stage of identification, event calculus is performed based on wide thresholds to identify all rendezvous events that may indicate illegal transshipment. After inputting contextual geographical information and the dynamic data of all ships within a certain time and space range, the spatial features calculation module calculates the ship’s geospatial features (intersections with special areas and proximity to other vessels). The geospatial features and ship dynamic data are then processed by an event calculus system, which uses defined basic rules along with wide thresholds. After the event calculation, preliminary information about suspected illegal transshipment behavior is obtained, including participants and time period.

In stage two of identification, suspicion levels are classified based on clustering results and traffic density features of all events identified in stage one. Inputting the event information obtained in the first stage, the behavior features calculation module computes features corresponding to the wide thresholds used in the first stage, i.e., encounter speed, inter-vessel distance, and encounter duration. Then, the unsupervised clustering model is utilized to obtain the different feature distribution combinations, and obtain the events that meet the basic characteristics of illegal transshipment. Furthermore, the traffic density surrounding the participating vessels is calculated by the traffic feature calculation module. The clustering results are further graded into three levels of suspicion: high, medium, and low, corresponding to the red, yellow, and pink trajectories in Figure 1.

### 2.2. Problem Formulation

The problem this paper tries to solve is to characterize the movement patterns of suspected illegal transshipment in different sea areas to identify illegal transshipment behavior. Specifically, the movement pattern of the behavior is generally defined as two vessels sailing at low speed or stopping and close to each other for a longer period in a non-traffic intensive area.

The model input is a set of dynamic data sent by all ships traveling in a certain area over a period of time, defined as below:

(1) $$D=\left\{D_{m_{1}},D_{m_{2}},\cdots ,D_{m_{k}}\right\},$$

where *m* is the MMSI (Maritime Mobile Service Identity) of the vessel, *k* is the number of ships in the dataset, *D* consists of $D_{m}$ which represents dynamic data sent by vessel *m*, described below:

(2) $$D_{m}=\left\{D_{m}^{t_{m}^{1}},D_{m}^{t_{m}^{2}},\cdots ,D_{m}^{t_{m}^{n_{m}}}\right\},$$

where $n_{m}$ is the total number of dynamic data sent by vessel *m*, *t* represents the timestamp that the dynamic data was generated, $D_{m}$ consisting of $D_{m}^{t}$, contains information about the motion information of the ship *m* at moment *t*, denoted as below:

(3) $$D_{m}^{t}=\left\{{lon}_{m}^{t},{lat}_{m}^{t},{cog}_{m}^{t},{sog}_{m}^{t}\right\},$$

where $lon_{m}^{t}$, $lat_{m}^{t}$, $cog_{m}^{t}$, $sog_{m}^{t}$ represent the geographical location (longitude and latitude), COG (Course over Ground), and SOG (Speed over Ground) of vessel *m* at time *t*, respectively.

As a result of the overall method, information about suspected illegal transit behavior is identified within the spatial-temporal range covered by the input dynamic information, referred to as *E*,

(4) $$E=\left\{m_{a},m_{b},t_{s},t_{e},s\right\},$$

where $m_{a}$, $m_{b}$ represent the vessels suspected of being involved in illegal transshipment, $m_{a},m_{b}\in \left\{m_{1},m_{2},\cdots ,m_{k}\right\}$, $m_{a}\neq m_{b}$, $t_{s}$ and $t_{e}$ refer to the starting and ending times of the event, and *s* indicates the suspicion degree of the event.

After input of the dynamic data for all ships *D*, the first stage of the method calculates each vessel’s motion and spatial characteristics, and then fed these features into a wide threshold-based event calculus system to recognize all rendezvous behaviors and determine the basic event information $\left\{m_{1},m_{2},t_{s},t_{e}\right\}$. In the second stage of the methods, based on the basic event information, event features are computed and used for unsupervised clustering, and then traffic flow characteristics near the vessel are combined to determine the degree of suspicion *s*, $s\epsilon \left\{High,\;Medium,\;Low\right\}$. Method details are provided below.

### 2.3. Event Calculus Based on Wide Threshold

This section is the first stage of the overall approach aiming to identify rendezvous events as comprehensively as possible. The input dynamic data is first pre-processed to meet the needs of further processing calculations. The processed dynamic data is then used to calculate spatial relationships, and lastly, the basic rules of rendezvous behaviors are used along with wide thresholds to obtain preliminary suspected illegal transshipment activities.

#### 2.3.1. Dynamic Data Pre-Processing

Dynamic data of vessels may contain abnormal values or missing values due to noise or incorrect coding and decoding, so the following steps are taken to prepare the dynamic data for calculation:

1. Removing messages that contain invalid data, including MMSI value is not 9 bits, SOG value beyond the set of [0–102.3], COG value beyond the set of [0–360], longitude value beyond the set of [−180–180], latitude value beyond the set of [−90–90];
2. Removing messages that contain missing data;
3. Remove vessel dynamic data if less than ten messages have been sent by the ship;
4. Sort dynamic data by timestamp in ascending order.

A very small number of messages is insufficient to calculate a trajectory that is representative of the characteristics of illegal transshipments. Since event calculus employs a sliding window processing mechanism, which means that a fixed step is moved forward to process data in the next fixed window, the behavior of the two vessels cannot be identified if their dynamic data does not fall into any window together, sorting by time will avoid this situation.

#### 2.3.2. Spatial Features Calculation

The transshipment occurs between ships that are in close proximity, and it is unlikely that strict supervision areas such as nearby ports, coastlines, and anchorages will be selected, so it is necessary to calculate these spatial characteristics between ships as well as between ships and special sea areas for event calculation. PostgreSQL database with PostGIS extension is used in this section.

Dynamic data may not be broadcast at the same time or frequency for each ship, resulting in misalignment of the location points despite the ships being physically close to each other. To calculate the proximity between two ships more accurately without disturbing the original data, a sub-trajectory $tra_{m}^{i}$ is generated between the position points of two consecutive dynamics data $D_{m}^{t_{i}}$ and $D_{m}^{t_{i+1}}$ of vessel *m*, where $i=1,2,\cdots ,n_{m}-1$. Each sub-trajectory $tra_{m}^{i}$ begins at $t_{m}^{i}$, ends at $t_{m}^{i+1}$, and all sub-trajectories form the trajectory library $\mathrm{Trajectory}y_{m}$ of vessel *m*, as described below:

(5) $${\mathrm{Trajectory}}_{m}=\left\{{tra}_{m}^{1},{tra}_{m}^{2},\cdots ,{tra}_{m}^{n_{m}-1}\right\},$$

If the difference between $tra_{m_{a}}^{i}$ and $tra_{m_{b}}^{i}$’s start time is less than $T_{d}$ seconds, they are considered to be generated simultaneously. $T_{d}$ is determined by the frequency of messages sent within the current data set. Then, calculate the distance between $tra_{m_{a}}^{i}$ and $tra_{m_{a}}^{i}$ as follow:

(6) $$d\left({tra}_{m_{a}}^{i},{tra}_{m_{b}}^{j}\right)=\;\mathrm{ST}\_\mathrm{DistanceCPA}\left({tra}_{m_{a}}^{i},{tra}_{m_{b}}^{j}\right),$$

ST_DistanceCPA is a built-in function in the PostGIS extension to calculate the distance between two trajectories at the nearest point. In the time intervals of the union of two trajectories, $m_{a}$ and $m_{b}$ are considered close if $d\left({tra}_{m_{a}}^{i},{tra}_{m_{b}}^{j}\right)$ is less than the distance threshold $E_{d}$.

The spatial relationship between the ship and the special area is calculated by the built-in function $\mathrm{ST}\_\mathrm{Contains}(\mathrm{geo}m_{A},\mathrm{geo}m_{B})$ in the PostGIS Geographic Information Extension Toolkit, which determines if the geometric structure B is contained in the geometric structure A. If *B* is fully enclosed in *A*, or if any points are located inside *A*, then it returns True. Otherwise, it returns False. As discussed in this paper, *A* is special sea areas that include near-coastal areas and near-port areas and *B* is the position points in the ship dynamic data. The area within 1 nautical mile from the shoreline is regarded as the near-coastal area, and the circular area with a radius of 1 nautical mile from the center of the port coordinates is regarded as the near-port area and the time of ships entering and leaving special sea areas is further determined for event calculation.

#### 2.3.3. Rendezvous Event Recognition

Illegal transshipment behavior is indicated by the low speed of two ships close to each other for a long time far from port and coastline, denoted as rendezvous events. Based on the dynamics and spatial characteristics of the vessel’s trajectory. The rendezvous event recognition approach used in this section is similar to the literature [17], but with different rules and thresholds.

Literature [17] defines rendezvous behavior as two vessels (excluding pilots and tugs) that remain within 100 m of each other and keep a speed of fewer than 5 knots for more than 4 min far from ports and coastline. (The original article does not specify the duration threshold, but the shortest duration in its published rendezvous recognition results is 241 s, indicating that it may use a 4 min duration threshold). The rules and thresholds defined in this paper differ from above in the following ways:

- Wider thresholds for event definitions;
- Vessel types are not restricted.

The wider threshold is designed to capture all suspected illegal transshipment behavior as broadly as possible, eliminating the problem of missed identification due to fixed thresholds, and then determining the most appropriate distribution combination of thresholds using unsupervised clustering methods in stage two.

The reason for not restricted vessel types is that the proportion of real illegal transshipment in the sea is usually very small, which is not enough to support the unsupervised clustering model to separate them, and the inclusion of tugboats, pilot boats, and other special types of vessels that need to perform legal transshipment can increase the proportion of real transshipment behavior. Thus, the ship type was not restricted in the model training phase, but they will be removed in the test stage and practical application.

The complexity and detailed analysis of the Composite Event Recognition and the reasoning algorithms of RTEC can be found in the literature [17,25]. After event calculation, preliminary information of each suspicious illegal transshipment is obtained:

(7) $$E=\left\{m_{a},m_{b},t_{s},t_{e}\right\},$$

During the spatial feature calculation stage, a vessel trajectory is divided into sub-trajectories between every two points, resulting in the splitting of one event into multiple ones. To restore the real duration of a single event, the segmented consecutive events must be merged. The merge logic is as follows: for all suspected illegal transshipment events involving the same two vessels, if a suspect event’s end time is not more than *I* minutes different from a suspected event’s beginning time, the two events are combined into one event, and the time interval is taken for the union set of the two events.

### 2.4. Classify Suspicion Levels Based on Clustering and Traffic Feature

The preliminary selection of suspected illegal transshipment events is based on a wide threshold to reduce missed identification, but many misidentification events are introduced simultaneously. In this section, to separate illegal transshipment behavior from others, the combination of threshold distributions applicable to the current sea area were determined by unsupervised clustering, and combined the traffic density feature to obtain the suspicion degree.

#### 2.4.1. Behavior Features Calculation

The thresholds associated with transshipment events, including vessel speed, the distance between two vessels, and event duration, have been selected as behavior features. To reflect the features of the event overall, the vessel speed and the distance between two vessels are taken as averages. The formula for calculating each feature of event *E* is as follows:

(8) $$\overset{-}{v_{1}}=\;\;\frac{1}{n}\sum_{i=1}^{n}{sog}_{m_{a}}^{i},\overset{-}{v_{2}}=\;\;\frac{1}{l}\sum_{j=1}^{l}{sog}_{m_{b}}^{j},$$

(9) $$\overset{-}{proximity}=\mathrm{geodesic}.\mathrm{WGS}84.\mathrm{Inverse}\left(\frac{1}{n}\sum_{i=1}^{n}{lon}_{m_{a}}^{i},\frac{1}{n}\sum_{i=1}^{n}{lat}_{m_{a}}^{i},\frac{1}{l}\sum_{j=1}^{l}{lon}_{m_{b}}^{j},\frac{1}{l}\sum_{j=1}^{l}{lat}_{m_{b}}^{j}\right),$$

(10) $$\mathrm{duration}=\;\;t_{e}-t_{s},$$

where *n* and *l* indicate the number of messages sent by $m_{a}$ and $m_{b}$ during the event, $\overset{-}{v_{1}}$, $\overset{-}{v_{2}}$ represent the average speed of the ship $m_{a}$ and $m_{b}$ in knots, $\overset{-}{proximity}$ denotes the distance between the average position points of the two ships, using the geodesic.WGS84.Inverse function in the Python toolkit Geographiclib to calculate the distance in meters, duration is the difference between the start and end time of the event in seconds.

#### 2.4.2. Clustering Modeling

Clustering is a method for analyzing data in an unsupervised manner to group similar data points together and discover underlying patterns, which has many applications such as market segmentation, image compression, and pattern recognition. To differentiate events characterized by illegal transshipment behavior from others in the absence of labeled data, we employed the K-means algorithm [26] which tries to partition the dataset iteratively into *k* pre-defined distinct non-overlapping clusters to provide classification results with high feature similarity for samples within clusters, and low feature similarity between clusters.

A summary of the K-means algorithm can be found in three main steps:

1. Set the number of clusters *k* and initialize the centroids by randomly selecting *k* data points without replacement;
2. Assign each data point to the closest centroid, and recalculate the centroids of each cluster by taking the average of all data points within the cluster;
3. Iterate step 2 until the centroids do not change.

Since this method is sensitive to outliers, so outliers whose feature values exceed upper and lower limits of normal values need to be removed from the training set. The upper and lower limits are denoted as:

(11) $$f_{L}=\;Q_{1}-\left(1.5*IQR\right),$$

(12) $$f_{H}=\;Q_{3}+\left(1.5*IQR\right),$$

where $Q_{1}$ represents the first quartile which is the median of the lower half of the dataset, $Q_{3}$ represents the third quartile which is the median of the upper half of the dataset, and IQR represents the distance between $Q_{3}$ and $Q_{1}$.

After removing outliers, each feature is then normalized to eliminate the influence of different features unit on the distance calculation when clustering. The transformation function between original data *x* and normalized data $x^{*}$ is

(13) $$x^{*}=\frac{x-\mu }{\sigma },$$

where mu and sigma represent the mean and standard deviation of all sample data.

Before training the K-means clustering model, it is necessary to determine the optimal number of classifications. Here, the elbow method is employed. The core idea of elbow methods is that when the number of clusters *k* is smaller than the true number of clusters, *k* increases will significantly increase the degree of aggregation of each cluster, which shows a large drop in clustering error SSE (Sum of the Squared Errors), we can compute:

(14) $$SSE=\sum_{i=1}^{k}\sum_{p\in C_{i}}{\left|p-m_{i}\right|}^{2},$$

where $C_{i}$ is the *i*th cluster, *p* is the data point in $C_{i}$, and $m_{i}$ is the centroid of $C_{i}$. When *k* reaches the proper number of clusters, the drop in SSE will rapidly become smaller, so the graph of SSE versus *k* is elbow-shaped, and the elbow corresponds to the optimal number of classifications for that sample. Afterward, the clustering model is trained using pre-processed training set data samples to divide data into a selected number of classes.

Then, using the validation set determine whether the previously trained clustering model was able to separate events characterized by illegal transshipment behavior (i.e., two ships sailing slowly and closer for longer periods), and if it did, the previously trained clustering model is used in the subsequent suspicion classification.

If the events cannot be separated well, this could indicate that there may be too few transit events compared to other normal navigation data or that the differentiation between transit behavior and other events in the current training set is not apparent. The classification effect can be optimized by adding more known transit events (which can be legal) to the training set or by expanding the threshold value during event calculation in the first stage.

#### 2.4.3. Traffic Density Feature Calculation

The existing identification rules can easily be satisfied by vessels moving normally in areas with heavy maritime traffic flows (e.g., ports, waterways, and major traffic routes), resulting in misidentifications. Additionally, illegal transshipment generally avoids these areas due to their stricter regulation. To minimize misidentification in further classification, a traffic density feature is introduced, denoted as $Tra_{D}$.

Traffic density is defined as the average number of vehicles that occupy one mile or one kilometer of road space, expressed in vehicles per mile or per kilometer. In our case, For event $E=\{m_{a},m_{b},t_{s},t_{e}\}$, the number of vessels that occurred in 1 nautical mile around the center point *C* during the event duration $\left.{\;\left[t\right.}_{s},t_{e}\right]$ been seen as the traffic density, denoted as $t_{d}$, where *C* is the average latitude and longitude of the ship $m_{a}$, the calculation formula is as follows:

(15) $$C=\left(\frac{1}{n}\sum_{i=1}^{n}{lon}_{m_{a}}^{i},\frac{1}{n}\sum_{i=1}^{n}{lat}_{m_{a}}^{i}\right),$$

The traffic density feature is the traffic density $td_{n}$ expressed in fuzzy variables categorized as sparse, medium, and dense denoted as follows:

(16) $${Tra}_{D}=\left\{\begin{array}{c} \;\;\;\mathrm{sparse},\;\;\;\;0<\;td_{n}\leq 1\;\;\\ \mathrm{moderate},\;\;\;\;1<\;td_{n}\leq 4\;\;\;\\ \mathrm{dense},\;\;\;\;\mathrm{others}\;\;\;\;\;\;\;\; \end{array}\right.$$

The traffic density feature of an event is considered sparse when $td_{n}$ is 1 during the event, indicating that there are no other vessels present besides the other ship involved in the event. A moderate traffic density is considered during an event when there are 2 to 4 ships around, while a dense traffic density is considered when there are more than 4 ships.

#### 2.4.4. Classify Suspicion Level

Combining the clustering results with the traffic feature to classify all suspected illegal transshipment events into three levels of suspicion $s,s\epsilon \left\{\mathrm{high},\;\mathrm{medium},\;\mathrm{low}\right\}$, as shown in Table 1.

The degree of each clustering feature in Table 1 is determined by comparing it with other categories in the classification results, and the actual regulatory requirements corresponding to each category are described below:

- High: The characteristics of suspected illegal transshipment are generally slow speed, close proximity, long duration, and the avoidance of areas of heavy traffic flow;
- Medium-1: Although the clustering features of this category meet the conditions, there are a few ships around, and a multi-vessel joint operation could be indicated by this observation. Meanwhile, when there are few ships in the surrounding waters, it remains very suspicious to approach at a low speed for a prolonged period;
- Medium-2: This type is in the case of no other ships nearby, but the two ships are slow and close, and the incident lasts only a relatively short period. This makes the transfer more difficult, but there is still the possibility of a transshipment taking place;
- Medium-3: This kind of event involves two relatively faster ships but at a close distance for a long time, and there are no other ships nearby. In certain circumstances, when the ships are sailing faster before approaching each other, the average speed is increased, and when no other ships are present, choosing to approach at high speed is not following normal safe navigation practices. So this situation is also classified as medium-suspicious;
- Low-1: This scenario is more like a misidentification event generated in a dense traffic sea area. Meet the characteristics of slow ship speed, close distance, and long time, but the number of surrounding ships is large;
- Low-others: The rest of the events have one or more of the following characteristics: fast speed, long distance between two ships, short event duration, or a large number of surrounding ships, which do not meet the general conditions of suspected illegal transshipment, and the suspicion of such events is classified as low suspicion.

## 3. Experimental Evaluation

To test the method’s performance in identifying illegal transshipment and its applicability to different sea areas, a publicly available AIS dataset [27] in France and a coastal regional AIS dataset in China are used. Based on the first dataset, we compare our results with the outcomes of rendezvous behavior [28] identified by the composite event recognition system proposed in [17], using rules and thresholds set based on expert experience. Based on the second dataset, as well as comparing the results with the method documented in the literature [17], the validity is further verified using real legal transshipment events and traffic flow sea areas.

### 3.1. Experimental Setup

#### 3.1.1. Dataset near the Port of Brest, France

**Figure 2:**
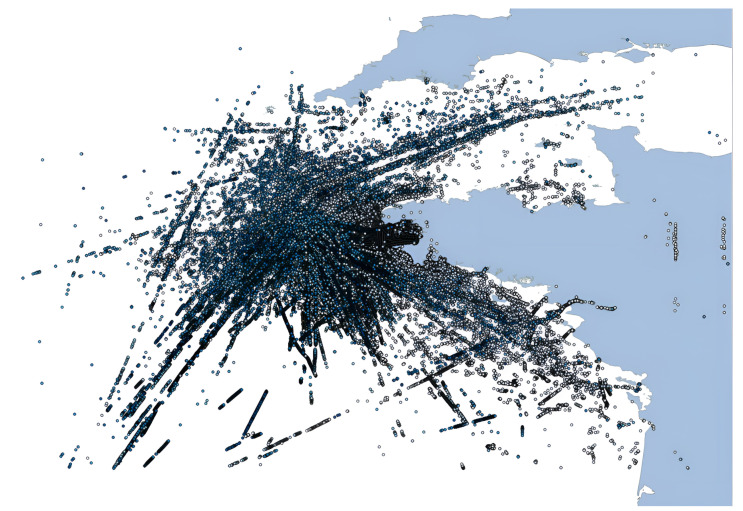
Vessel positions of the Brest, France dataset.

The dataset includes 18 M messages from 5 K ships sailing in the Atlantic Ocean near the port of Brest, France, with longitude coverage from −10° to 0°, latitude coverage from 45° to 51°, and a time frame of 6 months from 1 October 2015, to 1 April 2016 [29]. Figure 2 illustrates the geographical coverage of the datasets.

The dataset was divided as follows: the first four months (1 October 2015–31 January 2016) were used as the training set for building an unsupervised clustering model, and the events of the fifth month (1–28 February 2016) were used as the validation set, combining the clustering results and traffic density characteristics to classify the behavior suspicion level, and the events of the sixth month (1–31 March 2016) were used as the test set to compare results with the literature [17].

#### 3.1.2. Dataset near the port of Rizhao, China

**Figure 3:**
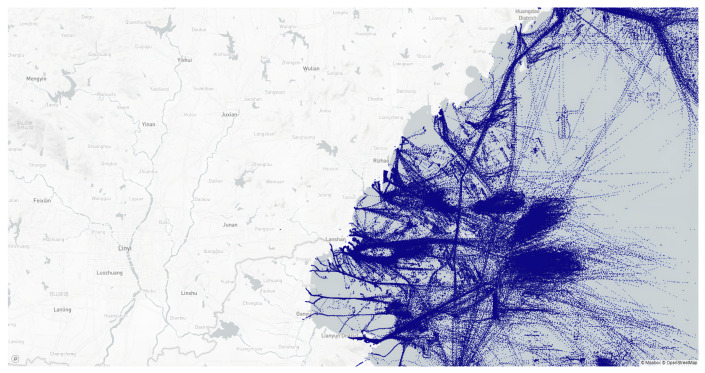
Vessel positions of the Rizhao, China dataset.

The dataset includes 33 M messages from 32 K ships sailing near the port of Rizhao, China, with longitude coverage from 118° to 120°, latitude coverage from 34° to 36°, and a time frame of 6 months from 1 January 2019 to 1 July 2019. This AIS dataset is collected by satellite with a message interval of about 200–400 s. Figure 3 illustrates the geographical coverage of the datasets.

In this dataset, from January to April 2019 is taken as the training set, the data from May 2019 as the validation set, and June as the test set. To verify whether the proposed method applies to different sea areas, the same experiments were conducted as in the Brest dataset. In addition, the ability to identify real transshipment behavior was verified by analyzing trajectories generated by legal transfers. Furthermore, the sea area with traffic flow characteristics in this dataset was used to verify the ability to discriminate against misidentification. The characteristics of the two datasets are compared in Table 2. Preliminary suspicious events are the result of the first stage of the methodology.

#### 3.1.3. Implementation Details

Table 3 lists the thresholds used in the first stage of our method and the literature [17]. For comparison, the method proposed in this paper will be referred to as the adaptive threshold method and the method in the literature [17] will be referred to as the fixed threshold method in the following.

The experiments were performed in Ubuntu 20.04.2 LTS. In the first stage of the method, spatial features calculus using PostgreSQL 12.8 database with PostGIS 3.1 extension, and event calculus using the open-source RTEC composite event recognition engine [25] as the literature [17], under SWI Prolog. In the second stage, the unsupervised clustering analysis was performed using Python 3.9.7 and the scikit-learn 1.0.2 package [30].

Experiments show that the clustering pattern stabilizes after the number of training sets reaches 10,000. In the following experiments, 10,000 randomly selected events from the training set were used to train the clustering model.

### 3.2. Result Comparison on the Brest Dataset

#### 3.2.1. Recognition Modeling

**Figure 4:**
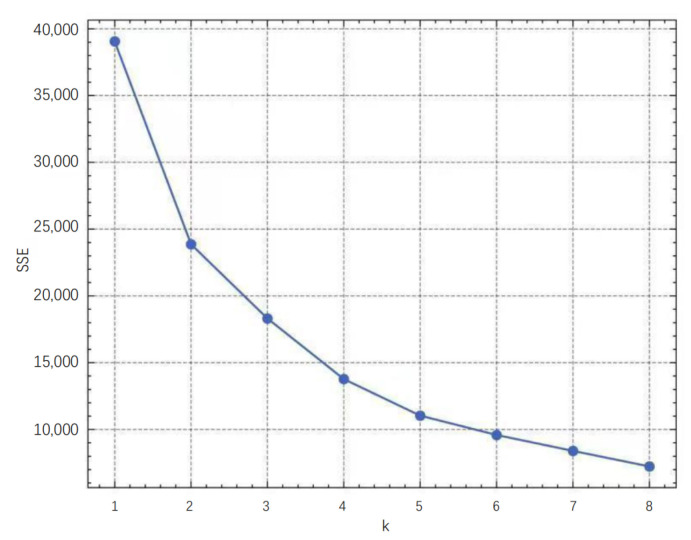
Line chart of the SSE for each value of *k*.

Within this dataset, 13 K preliminary suspected events involving 745 vessels were identified. After pre-processing with outlier removal and normalization, the clustering error SSE of the K-means model is calculated for each clustering number $k\in \left\{1,2,3,4,5,6,7,8,9\right\}$ to select the optimal number of clusters in the dataset. The relationship between SSE and *k* is shown in Figure 4.

According to the elbow method described in Section 2.4.2, if the line chart looks like an arm, then the “elbow” on the arm is the value of *k* that is the best. As shown in Figure 4, when *k* exceeds 4, the decreasing trend of SSE becomes relatively flat as compared with the previous trend, so 4 classes are selected as the optimal number of clusters for the training. Table 4 presents a description of each cluster derived from the unsupervised K-means clustering model.

In the clustering results, Class1 and Class4 are both sailing at a lower speed and in close proximity, but Class4 has a longer duration compared to Class1. Both Class2 and Class3 involve a slow and a fast ship sailing relatively far from each other for a long period, and the difference is the higher speed position (v1 or v2).

**Figure 5:**
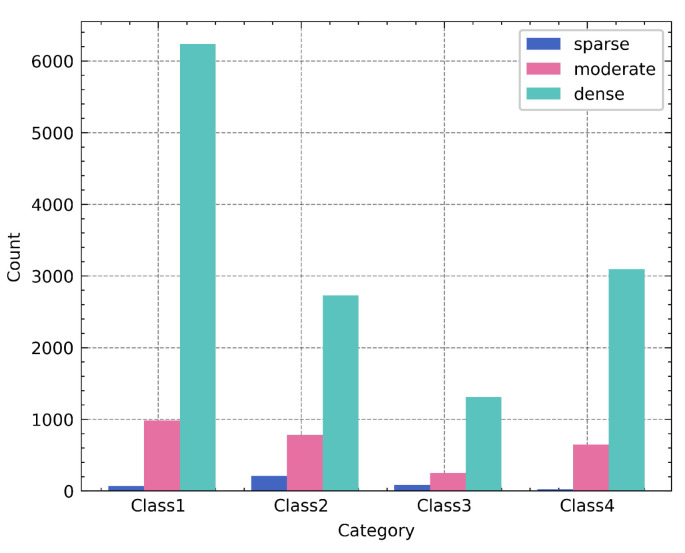
Traffic density feature for each category of the Brest, France validation dataset.

Despite categories 1 and 4 fitting the characteristics of suspected illegal transshipment behavior, their total number exceeded half of the total (72.86%). To further narrow the scope of suspicious events, the number of ships within 1 nautical mile was calculated as the traffic density feature, and the feature of each cluster in the validation set is shown in Figure 5.

Figure 5 shows that, although the total number of events in Classes 1 and 4 is higher, a smaller percentage of them occurred in areas with low traffic density, even less than the number of events in Classes 2 and 3.

By comparing the clustering and traffic features with Table 1, the events with categories 1, 4, and sparse traffic density correspond to High; the events with categories 1, 4, and moderate traffic density correspond to Medium-1; the events with categories 4 and dense traffic density correspond to Low-1; other events correspond to Low. The results of the suspicion degree grading of all events are shown in Table 5.

#### 3.2.2. Comparison of Identification Results

Based on the test dataset, after removing specific types of vessels (Tug, Pilot Vessel, Towing, Dredging or underwater ops, Law Enforcement, Search and Rescue vessel, Wing in groud, Medical Transport, Military ops), the identification results using the model established in Section 3.2.1 were compared to the open results of fixed threshold methods [28], as shown in Table 6.

According to the number of events in Table 6, the majority of primarily suspicious events involve low-suspicious events, and the number of highly suspicious events is similar to what would be expected using a fixed threshold. For low-level suspicious events, fewer ships generate a large number of ship-pair combinations and events, indicating that most of them have had suspicious interactions with several other ships. The number of vessels involved in all primary events is less than the sum of the three levels of suspicion, indicating that several vessels have generated different levels of suspicious events.

**Figure 6:**
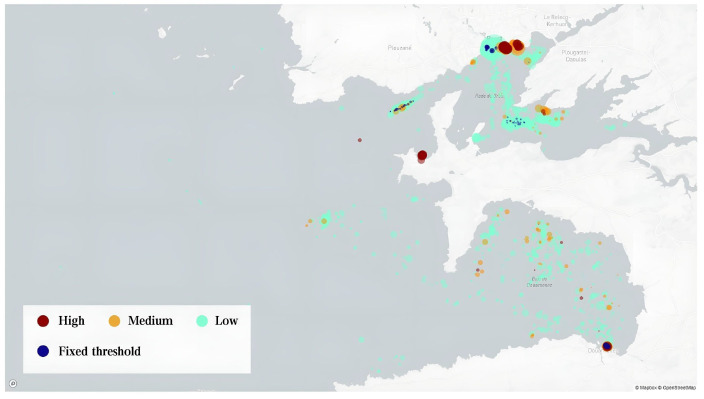
Visualization of suspected illegal transshipment identification results.

A visual representation of each type of event is provided in Figure 6, each bubble represents a suspected illegal transshipment event, its center represents the centroid of the overall trajectory, and its size represents its duration. The red, yellow, and sky blue colors of the bubbles correspond to high, medium, and low-suspicious events, respectively, and dark blue represents the events identified by the fixed threshold method.

Figure 6 shows that low-level suspicious events exhibit characteristics of traffic flow. Medium and high events are more scattered. There are some suspected illegal transshipments with long duration near the port, as well as the results of fixed threshold.

All events identified by the fixed threshold method are included in the results of our methods, and 127 events were identified earlier. In addition, the results identified by the fixed threshold method correspond to the degree of suspicion in our results as presented in Table 7.

Among the events classified as low-suspicious, 89 events had high traffic density, and the characteristics of the remaining events as shown in Table 8.

These six events are classified into 2 and 3 categories by using the clustering model, with a common characteristic of short duration (less than 10 min). Events No.1–5 are farther away on average proximity than event No.6, while the average vessel speed of event No.6 is relatively high during the short period.

**Figure 7:**
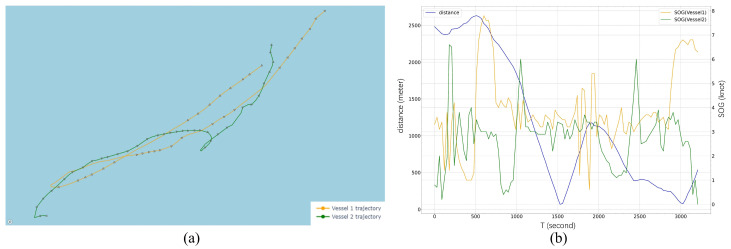
Highly suspicious events not identified by fixed threshold methods: (**a**) Trajectories of the involved vessels; (**b**) Change of distance and speed during the event.

Compared with the fixed threshold methods, 165 extra highly suspicious events involving different vessels were identified by our methods. An example is used to illustrate the reason why these events cannot be identified by the fixed threshold method. To analyze the trend before and after the event, the duration is extended by 1200 s from the original result, and the corresponding trajectories and the interaction characteristics of the involved vessels were shown in Figure 7.

Figure 7a depicts the trajectory of the two vessels, and the arrow indicates the COG at that point. At the beginning of the event, Vessel1 and Vessel2 are far away and traveling in opposite directions, and following the first intersection, both vessels start to adjust their course until the two ships are once again close to each other, thereafter keep sailing in the same direction. Figure 7b illustrates the corresponding changes in velocity and proximity of the two trajectories in Figure 7a, showing that the two vessels intersected with the same trend of velocity change around the 1500 s, and that they intersected again around the 3000 s. Since the two vessels are under dynamic navigation, the duration of distance less than 100 m does not exceed 240 s. The fixed threshold method is not able to identify them during the whole period.

**Figure 8:**
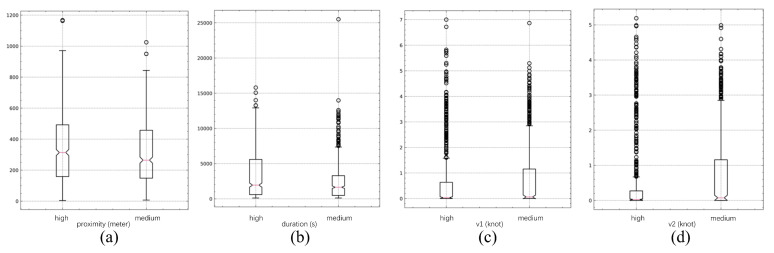
The high and medium-suspicious event features: (**a**) average proximity; (**b**) duration; (**c**) average speed of vessel 1; (**d**) average speed of vessel 2.

A box plot of all highly and moderately suspicious events is shown in Figure 8. This figure shows that, in general, the distance between the two vessels is close, the speed is slow, and the duration is long, which is consistent with the basic characteristics of suspected illegal transshipment activities.

### 3.3. Result Comparison and Identification Ability Validation on the Rizhao Dataset

#### 3.3.1. Recognition Modeling

In this dataset, the same methods, thresholds, and operating environment are used as in Section 3.2.1. After the first stage of the method calculation, 355K preliminary suspected events involving 12K vessels were identified in 6 months. Following the random selection of 10,000 samples from the training set, removing the outliers, and normalizing them, four clusters were selected using the elbow method. The training results of the clustering model are presented in Table 9.

**Figure 9:**
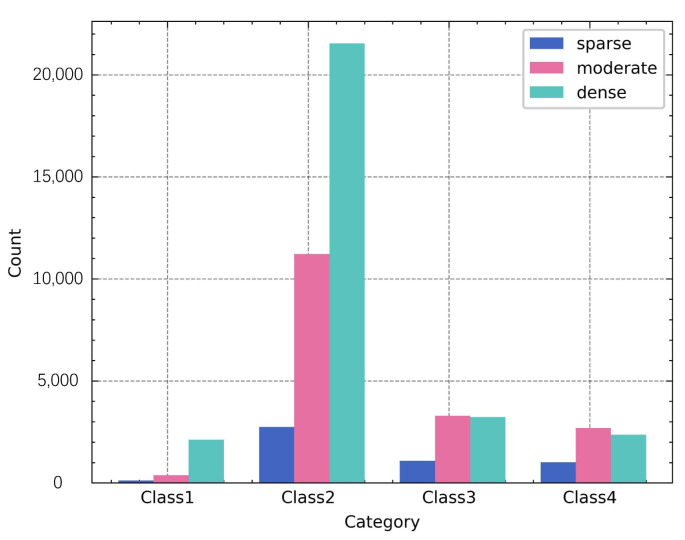
Traffic density feature for each category of the Rizhao, China dataset.

In the clustering results, category 2 has a relatively low ship speed, category 1 has a longer event duration, category 3 has a faster speed, and category 4 has a larger average distance between the two ships. Figure 9 shows the traffic density for each cluster.

In Figure 9, the majority of events in category 2 are generated with dense traffic density, while the proportion of events with dense traffic density in categories 3 and 4 (higher speed) is relatively low in comparison to categories 1 and 2 (slower speed).

By comparing the clustering and traffic features with Table 1, the events with categories 1 and sparse traffic density correspond to High; the events with categories 1 and moderate traffic density correspond to Medium-1; the events with categories 2 and sparse traffic density correspond to Medium-2; the events with categories 1 and dense traffic density correspond to Low-1; other events correspond to Low. The results of the suspicious degree grading of all events are shown in Table 10.

#### 3.3.2. Comparison with Fixed Threshold Method

Based on the test dataset, after removing specific types of vessels, the identification results using the model established in Section 3.3.1 were compared to the fixed threshold method using the same thresholds, as shown in Table 11.

**Figure 10:**
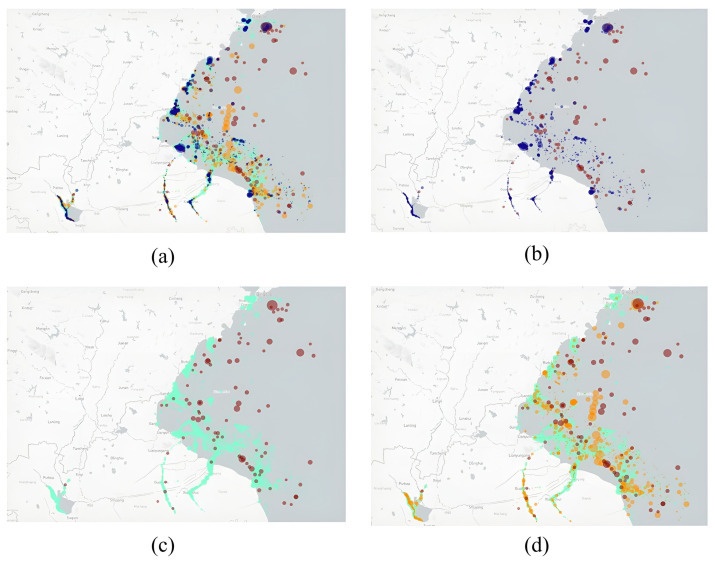
Visualization of suspected illegal transshipment identification results: (**a**) High/Medium/Low/Fixed threshold; (**b**) High/Fixed threshold; (**c**) High/Low; (**d**) High/Medium/Low (High-red bubble, Medium-yellow bubble, Low-sky blue bubble, Fixed threshold-dark blue bubble).

In this dataset, the fixed-threshold approach identified far more events than the methods presented in this paper considered highly suspicious. The event visualization can be seen in Figure 10, the high, medium, and low suspicion results are shown in red, yellow, and sky blue colors, respectively, while the result of the fixed thresholds method is displayed in dark blue, with the bubble size representing the duration of the event.

Figure 10a shows the identification results of our method compared to those obtained by the fixed threshold method, and it encompasses the latter in its geographical spread. Figure 10b shows that the fixed threshold methods produce a lot of results along the shoreline and in inland waterways, while the highly suspicious events are mainly clustered in areas that are remote from the shoreline. The same trend is observed in the comparison between high and low-suspicious events in Figure 10c. Figure 10d shows all suspicious events identified in this study. The dispersion of the geographical spread is positively correlated to the degree of suspicion.

Table 12 shows the results identified by the fixed threshold method correspond to the degree of suspicion in our results.

As shown in Table 12, the majority of fixed threshold methods results are classified as low suspicion, among which 55.73% have a high surrounding density of traffic, 29.78% have a medium surrounding traffic density but a short duration of event generation, and 3% have a relatively faster average speed during the short event period.

#### 3.3.3. Legal Transshipment Events

**Figure 11:**
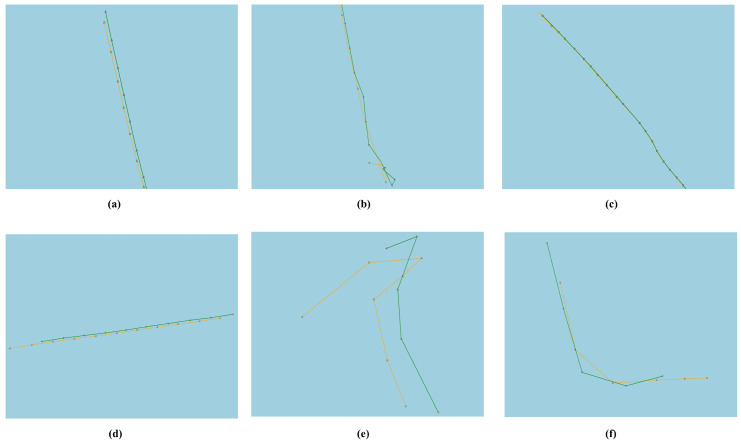
The trajectory of legal transshipment events, (**a**–**f**) correspond to events No. 1–6.

To verify the method’s ability to identify the real transshipment behavior and to classify the level of suspicion according to pre-designed criteria, six legal transfer events have different event characteristics were selected. Their trajectories are shown in Figure 11. These six events are all transshipment events involving tugboats, which are not included in the identification results of Section 3.3.2 and have not participated in clustering model training. Table 13 shows their motion characteristics, clustering results, traffic density feature, and the suspicion level of the identification using the methods in this paper.

All six events are identified by our method, and only one event was classified as low-suspicious due to the short duration and the presence of other vessels in the vicinity. Among the events considered as highly suspicious, all events were classified as category 1 by the clustering model for long durations, low average speed, and comparatively close distance, with no other ships around. Among the events considered as medium-suspicious, the events were all classified into category 2 for relatively shorter event durations, with no other ships around.

Event 6 was considered as low-suspicious due to the presence of a small number of other ships in the vicinity and the relatively short duration. It may be attributed to the fact that legal transshipment behavior in which ships have informed the local maritime authorities in advance for reasons such as difficulties reaching the destination directly, unlike illegal transshipment, usually occurs near ports, where traffic volumes are usually higher, and need not the circumvention of maritime surveillance.

In particular, No.2 and 3 events will be filtered out by fixed thresholds rule-based methods if the speed thresholds were set as 5 knots as in previous experiments.

#### 3.3.4. Sea Areas with Traffic Flow Characteristics

**Figure 12:**
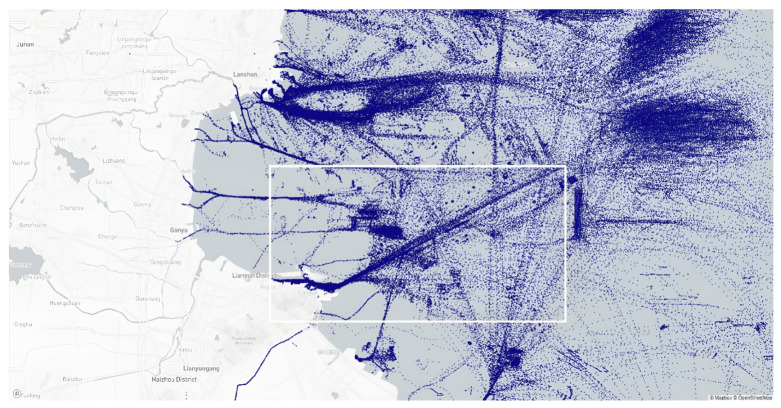
Sea area with obvious traffic flow characteristics.

To test the ability of the proposed method to discriminate misidentified behavior within traffic flows, the sea area with obvious traffic flow characteristics in this dataset, as shown in the area inside the white border of Figure 12, was selected, with a latitude range of [34.68–34.96] degree and a longitude range of [119.36–120.00] degree from 1 June 2019, to 10 June 2019.

Table 14 provides information on suspected illegal transshipment identified in the region within 10 days.

**Figure 13:**
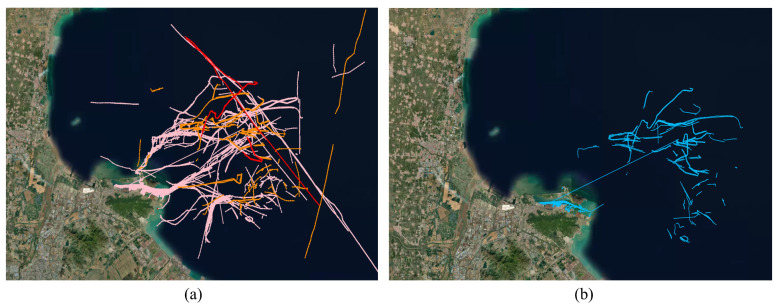
The trajectories of suspected events in traffic flow area: (**a**) adaptive threshold method (High-red line, Medium-yellow line, Low-pink line); (**b**) Fixed threshold method.

During 10 days, 65 highly and moderately suspicious events were identified in the region, representing a relatively small percentage of all reported. The trajectories of events at each level of suspicion are shown in Figure 13.

In Figure 13a, it can be seen that most of the trajectories in the main traffic flow areas in the diagonal direction are classified in the low-suspicious category (pink trajectories), and their trajectory is more consistent, mainly radially with the port as the origin. In contrast to the low-suspicious events, the high and medium-suspicious events have a more chaotic trajectory, and are generally located farther from the coast. Additionally, medium-suspicious events have a relatively shorter trajectory than high-suspicious events.

At the same time, the fixed thresholds methods identified 312 events in the same area, their trajectories are shown in Figure 13b. It can be seen that the recognition result partially overlaps with the traffic flow area and generates a lot of false recognition in the inland river area.

## 4. Discussion

The identification results in two different sea areas as shown in Table 6 and Table 11 demonstrate that our methods can detect more suspicious events than fixed threshold results, and according to Figure 8, these events with high and medium suspicion satisfy behavior features of illegal transshipment. There are several possible explanations for this result. Firstly, if the ships involved in the case continue to move during the process, the event that strictly meets the specified threshold may be divided, resulting in a short duration of a single event and causing the identification to be missed, as shown in Figure 7. Secondly, since the position broadcast by the vessel comes from the position where the sensor is located, the size of the ship will make the actual distance larger than the calculated one, so a small distance threshold will tend to filter out events related to large vessels. Thirdly, some transshipment behavior exhibits motion characteristics that exceed the fixed threshold, some real-world examples can be found in Section 3.3.3.

By analyzing the result of fixed threshold methods exhibited in Table 7 and Table 12 in the two datasets, we found about 61.38% and 80.13% of the identifications are generated in areas with greater traffic density, respectively. From the geographical distribution of identification in Figure 6, Figure 10 and Figure 13, the highly and moderately suspicious events identified in this paper turned out to be more distant from strictly regulated areas such as coasts and ports. This indicates that fixed threshold approaches without taking into account the traffic density characteristics are likely to lead to a large number of false positives in dense marine areas without predefined geometry. This was further verified in Section 3.3.4, based on the sea area with obvious traffic flow, most of the events occurring within the traffic flow area are classified as low suspicion by our methods, and the number of high and medium-suspicious events within 10 days remains within the supervisory range, which meets the practical application requirements, and the method based on fixed thresholds generates a large number of suspected events in traffic dense area.

By comparing the clustering models of the two sea areas as in Table 4 and Table 9, as the sea area changes, the feature distribution of the clustering model also changes, still separating events that match the characteristics of illegal transshipment with stable proportions (Table 5 and Table 10), while the method with fixed thresholds produces a large number of false identification events in the densely trafficked Chinese coastal region, and with tests on the legal transshipment events, the clustering model can divide events as expected in Table 1.

## 5. Conclusions

This paper proposes a method to identify suspected illegal transshipment. First of all, this method combines a rule-based method with an unsupervised learning method, which can automatically determine the threshold distribution combination suitable for regulatory sea areas. It solves the problem of missing identification of fixed threshold method based on expert experience. Secondly, the method introduces traffic density features in the sea area, which can effectively reduce the false identification rate of suspected illegal transshipment. This method is validated on two sea areas. The results of French Breast port dataset show that this method can fully cover illegal transshipment events identified by the fixed threshold method.The results can be divided according to the suspicious degree and interpretable. Compared with the fixed threshold method, this method identifies more illegal transshipment events, which are highly suspicious, and gives warning much earlier. The proposed method can even filter out misidentification events from compared methods’ results, which account for more than half of the total number. The results of Chinese Rizhao port dataset show that this method can cover all illegal transshipment events identified by the fixed threshold method, which is consistent with the results of French breast port dataset. This method can effectively solve the problem of misidentification in dense traffic areas and improve the recognition accuracy. What’s more, it can identify all legal transshipment events. The proposed method is an active exploration in the application of the combination of rule-based method and machine learning method, which is significant for the practical application of identifying suspected illegal transshipment behavior.

The following three aspects shall be improved in the future. Firstly, different event features, such as the closest distance between vessels’ trajectories shall be considered to better distinguish illegal transshipment events. Next, limiting factors, such as AIS closures, can be taking into account to improve practical use. Last but not least, background information can be investigated to help further judge illegal transshipment from suspected illegal transshipment.

## Figures and Tables

**Table 1:** Classification of suspicion level.

Suspicion Level	Clustering Features			Traffic Density
	Velocity	Proximity	Duration	
High	slower	closer	longer	sparse
Medium	slower	closer	longer	moderate
	slower	closer	shorter	sparse
	slightly faster	closer	longer	sparse
Low	slower	closer	longer	dense
	Others			

**Table 2:** Datasets’ characteristics.

Attribute	Brest	Rizhao
Period(months)	6	6
Vessels	5 K	32 K
Position signals	18 M	33 M
Longitude	(−10°, 0°)	(118°, 120°)
Latitude	(45°, 51°)	(34°, 36°)
Preliminary Suspicious Events	13 K	155 K

**Table 3:** Thresholds used in the experiments.

	Thresholds	$T_{d}$	$E_{t}$	$E_{v}$	$E_{d}$	*I*
Methods						
Adaptive threshold (first stage)		80 s	1 min	15 knot	500 m	30 min
Fixed threshold		-	4 min	5 knot	100 m	-

**Table 4:** Unsupervised clustering model of Brest, France dataset.

Category	Behavior Features Distribution (mean/std)				Percentage
	v1 (knot)	v2 (knot)	Duration (knot)	Proximity (m)	
Class1	0.41/0.40	0.27/0.30	2142.73/0.51	288.56/0.36	55.36%
Class2	3.00/1.05	6.28/0.93	1137.15/0.38	835.61/1.20	18.65%
Class3	7.76/0.93	0.58/0.55	959.84/0.42	1171.63/1.21	8.49%
Class4	0.20/0.31	0.12/0.21	9162.87/0.85	275.51/0.33	17.50%

**Table 5:** Classify suspicion level for Brest, France validation dataset.

	High	Medium	Low
Category-Traffic density	Class4-sparse	Class4-medium	Others
	Class1-sparse	Class1-medium	
No. of events	92	1632	14,949
No. of vessels involved	66	118	212
Percentage	0.55%	9.79%	89.66%

**Table 6:** Result comparison in Brest, France test dataset.

	Adaptive Threshold				Fixed Threshold
	Primarily	High	Medium	Low	
No. of events	17,056	175	757	16,124	141
No. of vessel pairs involved	2944	64	241	1923	78
No. of vessels involved	241	75	107	229	59

**Table 7:** Suspicion level of fixed threshold identification results.

	High	Medium	Low
Fixed threshold method	10	31	95

**Table 8:** Fixed threshold results for non-traffic intensive, low-suspicious events.

No.	Duration (s)	Proximity (m)	v1 (knot)	v2 (knot)	$td_{n}$	Category
1	475	743.71	4.83	0.31	1	3
2	252	783.80	0.02	8.03	4	2
3	452	420.58	2.49	5.09	1	2
4	586	433.85	3.51	2.93	3	2
5	336	513.80	3.00	3.29	3	2
6	290	98.98	5.39	3.80	3	2

**Table 9:** Unsupervised clustering model of Rizhao, China dataset.

Category	Behavior Features Distribution (Mean/Std)				Percentage
	v1 (knot)	v2 (knot)	Duration (knot)	Proximity (m)	
Class1	5.71/0.84	5.73/0.84	4849.20/1.53	489.56/0.86	7.03%
Class2	3.23/0.59	3.17/0.58	473.66/0.47	559.39/0.65	60.33%
Class3	7.88/0.63	7.63/0.69	550.89/0.55	531.98/0.59	31.82%
Class4	6.38/0.97	6.97/0.91	348.48/0.45	1460.31/1.23	16.06%

**Table 10:** Classify suspicion level for Rizhao, China validation dataset.

	High	Medium	Low
Category-Traffic density	Class1-sparse	Class1-moderate	Others
		Class2-sparse	
No. of events	127	3142	50,043
No. of vessels involved	135	2676	8371
Percentage	0.24%	5.89%	93.87%

**Table 11:** Result comparison in Rizhao, China test dataset.

	Adaptive Threshold				Fixed Threshold
	Primarily	High	Medium	Low	
No. of events	45,026	99	2325	42,602	5487
No. of vessel pairs involved	30,584	54	1769	30,584	3404
No. of vessels involved	7344	106	2261	7344	3353

**Table 12:** Suspicion level of fixed threshold identification results.

	High	Medium	Low
Fixed threshold method	10	335	5142

**Table 13:** The features and identification results of legal transshipment events.

No.	Duration (s)	Proximity (m)	v1 (knot)	v2 (knot)	$td_{n}$	Category	s
1	3380	447.41	3.94	3.93	1	1	high
2	6446	139.92	6.50	6.58	1	1	high
3	2810	720.87	6.58	5.10	1	1	high
4	1831	347.67	4.84	4.77	1	2	medium
5	608	33.86	2.46	2.80	1	2	medium
6	286	304.15	2.90	2.85	3	2	low

**Table 14:** Identification results in the sea area with dense traffic.

	Adaptive Threshold				Fixed Threshold
	Primarily	High	Medium	Low	
No. of events	824	5	60	759	312
No. of vessel pairs involved	519	4	36	501	196
No. of vessels involved	283	8	60	269	177

## Data Availability

The data used to support the findings of this study are available from the first author upon request.
